# Frequency-doubled chirped-pulse dual-comb generation in the near-UV: combined vs separated beam investigations of Rb atoms and NO_2_ near 420 nm

**DOI:** 10.1038/s41598-025-00684-1

**Published:** 2025-05-25

**Authors:** Jasper R. Stroud, David F. Plusquellic

**Affiliations:** National Institute of Standards and Technology, Boulder, CO 80305 USA

**Keywords:** Chirped pulse, Dual electro-optic comb, Ultraviolet combs, Rubidium atom sensing, Nitrogen dioxide detection, Nonlinear optics, Imaging and sensing

## Abstract

We describe an electro-optic dual-comb system that operates in the near-infrared (near-IR) region to generate optical frequency combs in the near-UV by sum frequency generation in two configurations. The near-IR frequency combs are generated using chirped pulses that down convert the optical information into the radio frequency (RF) domain by a difference in the chirp bandwidths. Near-UV combs at twice the near-IR bandwidth are obtained by sum frequency generation in a nonlinear crystal and detected by a hybrid photon counting detection system. We compare the results of studies of Rb near 420 nm using two optical arrangements where the near-IR combs are mixed in the crystal as combined or as separated beams. While the latter method enables phase retrievals, the combined beam method is superior for phase stability, power throughput for detection, ease of alignment and detection sensitivity. High order interleaving enables near-UV bandwidths near 4 cm^−1^ for faint photonic sensing and spectroscopic applications. We demonstrate its utility as a high-resolution instrument for column detection of the toxic pollutant, NO_2_. The harmonic generation method is easily extendable across much of the titanium sapphire tuning range for detection of other trace gases.

## Introduction

The ultraviolet (UV) to visible region of the electromagnetic spectrum has provided vital information for key advances in diverse frontier areas that range from atom-based devices for optical frequency clocks^1^, to Rydberg atom sensing of magnetic^2^ and electric fields^3^, to the emerging fields of quantum sensing^4,5^ and imaging^6^. For an increasing number of these applications, optical frequency combs (OFC) provide easy access to the quantum states of interest and a robust route to the SI traceability of the spectroscopic measurements.

Mode-locked (ML) OFC methods have been transformational by providing high resolution spectroscopic signatures over multiple THz of optical bandwidth^7–9^. This technique has enabled many advances in trace gas detection in the near-infrared (IR) region and beyond^10,11^. The high peak power of the pulse train allows for efficient nonlinear frequency conversion across wide spectral regions from the microwave (MW) to vacuum UV (VUV) regions. However, for continuous wave (CW) generation in the UV region, the nonlinear mixing is primarily limited to frequency doubling in enhancement cavities in the 500 nm to 900 nm region^12–14^. For integrated photonic systems, sum frequency generation^15–18^ has been demonstrated into the visible range using lithium niobate (LiNbO_3_) electro-optic (EO) modulators and ring resonators. However, generating OFCs at shorter wavelength below 500 nm becomes increasingly difficult because of strong material dispersion and propagation losses. Moreover, for integrated LN devices, photorefractive effects from trapped charges have led to unacceptable phase drifts^19^ although recent devices without this drawback have been reported across the visible region from 400 to 700 nm^20^.

The region for interrogation of many atom-based devices is bounded to the tens of GHz range where the high power per comb tooth and the low relative phase noise of CW EO combs are well suited. Further, many other applications do not require THz of bandwidth provided by ML-OFCs, such as pressure sensing of a GHz wide spectra feature or tracking the position of a cavity resonance over tens of GHz for temperature or acceleration sensing^21–23^. Dual EO-OFCs are often limited in optical bandwidth but make up for it in hardware simplicity^24,25^. Using either one or two non-phase locked optical sources, the mutual phase coherence of the two legs of an interferometer with EOs has been shown to provide excellent phase stability without complicated phase locking setups. These components also can be easily integrated for deployment in on-chip devices^26^.

These attributes have made dual EO-OFC spectroscopy an invaluable tool in the infrared regions, but the lack of phase modulators in the UV region has made it difficult to directly implement this technique. The UV region is readily reached using nonlinear mixing processes that double near-IR photons into the UV in a nonlinear crystal^27^, but the conversion efficiency is often too low for traditional detection. Even for ML-OFC methods, direct comb generation below 500 nm in the visible requires a comb-pumped nonlinear mixing process either with type 0, 1 or 2 mixing in nonlinear crystals^28–32^ or for the VUV region, enhancement cavities together with noble gas jets^33–35^ or other solid materials^36,37^.

We have previously reported on using photon counting to accumulate interferograms for remote sensing in the near-IR region to achieve heterodyne detection at very low light levels^38^. In this article, we extend this dual comb technique into the near-UV region in combination with our differential chirped-pulse down-conversion technique^39–42^. A UV dual comb system has been recently reported that generates, in two stages, harmonic combs using a traditional dual EO-OFC method operating near 1.5 μm^29^. In this work, we use a CW source near 840 nm and dual chirped pulses of an EO-OFC to generate UV combs in a single stage and in a single crystal.

Chirped pulse spectroscopy is now routinely used in microwave region^43–46^, but is an emerging technique being applied as an OFC method^47^. Phase coherent repeated chirps generate flat frequency combs with comb spacings, and spectral bandwidths determined solely by the programable chirp parameters^48^. This allows for high flexibility of the desired optical resolution, bandwidth, and recording time, depending on the sensing requirements of the system. We have recently described a dual comb technique to extend the bandwidth for spectroscopy far beyond the detection bandwidth of the system, allowing for an optical region of > 100 GHz region to be recorded within an RF detection bandwidth of < 500 MHz in the near-IR^39^ and < 1 MHz^40^ in the THz region. Here, we extend this technique in the near-UV region where molecular structure, reaction dynamics and trace gas sensing can be performed at high-resolution.

## Experiment

### Combined beam (CB) and separate beam (SB) system configurations

**Fig. 1:**
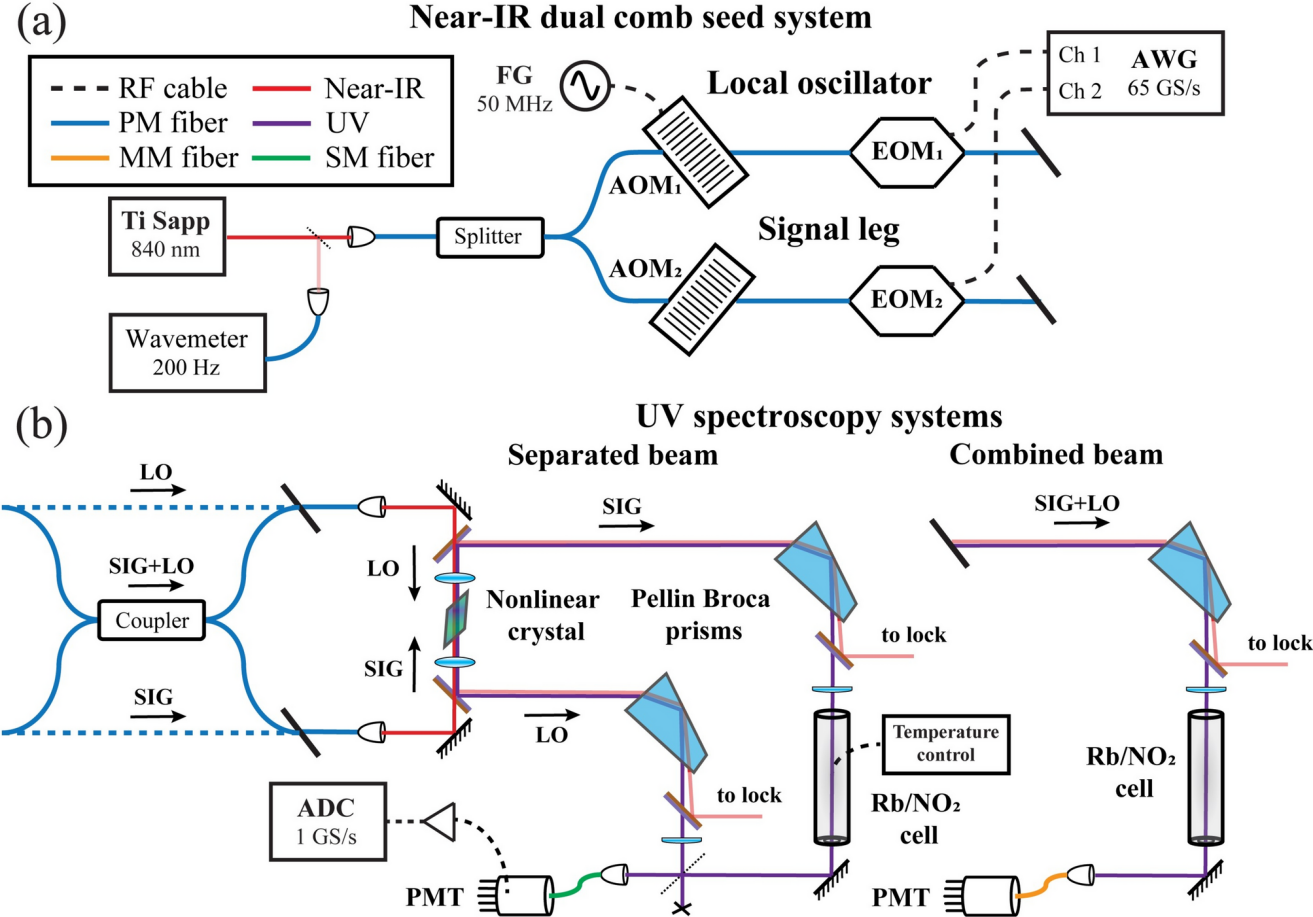
System diagram. (**a**) The common elements of the dual comb seed system. (**b**) UV generation for the CB and SB configurations showing the nonlinear mixing products are first dispersively filtered from the near-IR pump light using Pellin Broca prisms before passing through the sample cell and being detected by a PMT (AWG: arbitrary waveform generator, FG: function generator, AOM: acousto-optic modulator, EOM: electro-optic modulator, PMT: photomultiplier tube).

The experimental setup used to generate dual OFCs is shown in Fig. 1a. The chirped pulse waveforms define the OFC properties where the chirp duration is inversely related to the comb tooth spacing, the chirped interval defines the comb bandwidth, and the drive voltage defines the Bessel function distribution of EOM sideband amplitudes^39^. The two chirped pulses in the SIG and LO legs are defined to have the same chirp duration but different chirp bandwidths. The bandwidth difference defines the down-converted bandwidth in the radio frequency (RF) domain, $\Delta {f}_{RF}$,

1 $$\Delta {f}_{RF}=\Delta {f}_{LO}-\Delta {f}_{SIG}$$

where $\Delta {f}_{SIG}$ and $\Delta {f}_{LO}$ are the SIG and LO bandwidths, respectively. The different bandwidths also result in different chirp rates,

2 $${\alpha }_{SIG/LO}=\frac{\Delta {f}_{SIG/LO}}{{\tau }_{CP}}$$

For this work, the SIG chirp is defined from *f*_*SIGstart*_ = 1 GHz to *f*_*SIGstop*_ = 15 GHz and *τ*_*CP*_ = 50 μs duration. The LO chirp is defined from *f*_*LOstart*_ = *f*_*SIGstart*_ − *f*_*RFstart*_ to *f*_*LOstop*_ = *f*_*SIGstop*_ − *f*_*RFstop*_, where *f*_*RFstart*_ = 3 MHz and *f*_*RFstop*_ = 5 MHz. Therefore, the system down converts 14 GHz of optical bandwidth in each sideband to Δ*f*_*RF*_ = 2 MHz at the detector.

Two different arrangements are explored to generate near-UV combs near 420 nm by Type I phase matching inside a Brewster-cut lithium triborate (LBO) crystal (LiB_3_O_5_, 10 mm long, φ = 27.9°, θ = 90° for P-polarized 840 nm light, Conex Optics, Inc). In the first arrangement shown in Fig. 1b referred as the combined beam (CB) method, the two near-IR legs are first combined in a 50/50 fiber coupler and one of output legs is collimated and focused inside the LBO crystal with a f = 5 cm achromatic lens (f = focal length). In the second arrangement, also shown in Fig. 1b and referred as the separated beam (SB) method, the SIG and LO UV beams are generated separately in a crossed path configuration in a single LBO crystal where the SIG beam travels in one direction through the crystal, while the LO beam travels in the opposite direction. The arrangement simplifies the tuning of the system by the simultaneous Type I angle phase matching. This type of SB method has been discussed previously although doubling was performed in two crystals^29^.

## Near-UV comb generation

### Nonlinear mixing of dual chirped pulses

**Fig. 2:**
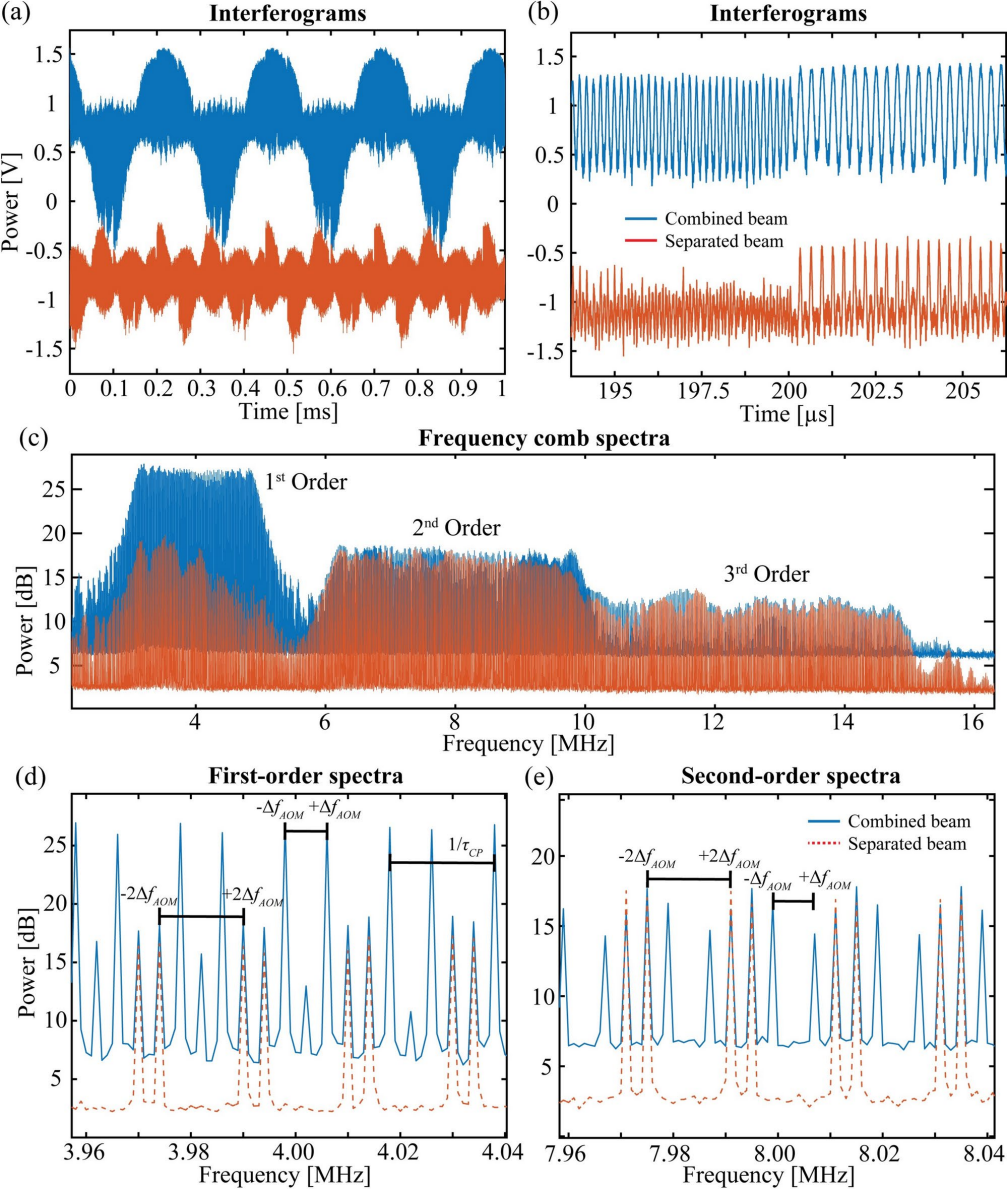
CB and SB interferogram and combs. (**a**) The interferogram obtained from the CB system in blue and the SB system in red, with (**b**) the zoomed in section showing the transition between chirped pulses. (**c**) The comb spectrum, with (**d**) the first-order comb lines and (**e**) the second-order comb lines. The corresponding SB data containing only frequency doubled comb lines is shown in red and offset for clarity.

The near-UV interferograms averaged over 15 min periods are shown in Fig. 2a for the SB (blue, top panel) and CB (red, bottom panel) methods. For the CB method, the 4 kHz beat note (i.e., four repeated patterns) is readily apparent, while for the SB case, two interleaved oscillations are observed at 8 kHz. Figure 2b shows an expanded portion of the interferogram in Fig. 2a near where a change in frequency occurs from the end of one chirp to the start of the next. The additional features in the CB interferogram relative to the SB response are due to the extra mixing products between the SIG and LO chirps that are produced in the nonlinear conversion in the crystal. Figure 2c shows the different orders of the comb spectra from the CB system in blue, and the SB system in red (offset for clarity). The second-order combs described by the second harmonic terms are shown from 6 to 10 MHz in Fig. 2c. The first-order and third-order combs shown in Fig. 2c are described by the mixing between the zero-order and first-order, and first-order and second-order components, respectively. Figure 2d and e show expanded portions of the first-order and second-order spectra, respectively, where lines appear separated by twice the AOM beat frequency in both configurations.

As discussed in Methods, the second harmonic and the inter-order mixing products describe all the comb spectra generated by the SB system and are shown in red in Fig. 2c, d and e. In contrast, the results for the CB system (in blue) clearly have several additional combs. These added features are due to the mixing between the SIG and LO chirps that generate UV combs but with only a single AOM beat note shift.

Further, these products form combs (designated degenerate combs) that are not unique in the RF domain because multiple optical spectral regions are mapped onto the same RF comb. (In the supplemental materials, we illustrate how the degenerate combs map the fringes of a high finesse etalon at 420 nm.) However, as shown in Fig. 2d and e, the degenerate combs are cleanly separated from the uniquely mapped ones because of the differences in the beat frequencies. While both methods produce uniquely mapped spectra, the SB method produces a far cleaner spectrum and allows for more possible combinations of beat note differences and interleaved EOM orders for the generation of different spectral resolutions and bandwidths. As a case in point, the degenerate comb begins to overlap with the unique fifth-order comb with the system settings used in this work (4 kHz beat note, 50 µs chirp duration).

**Fig. 3:**
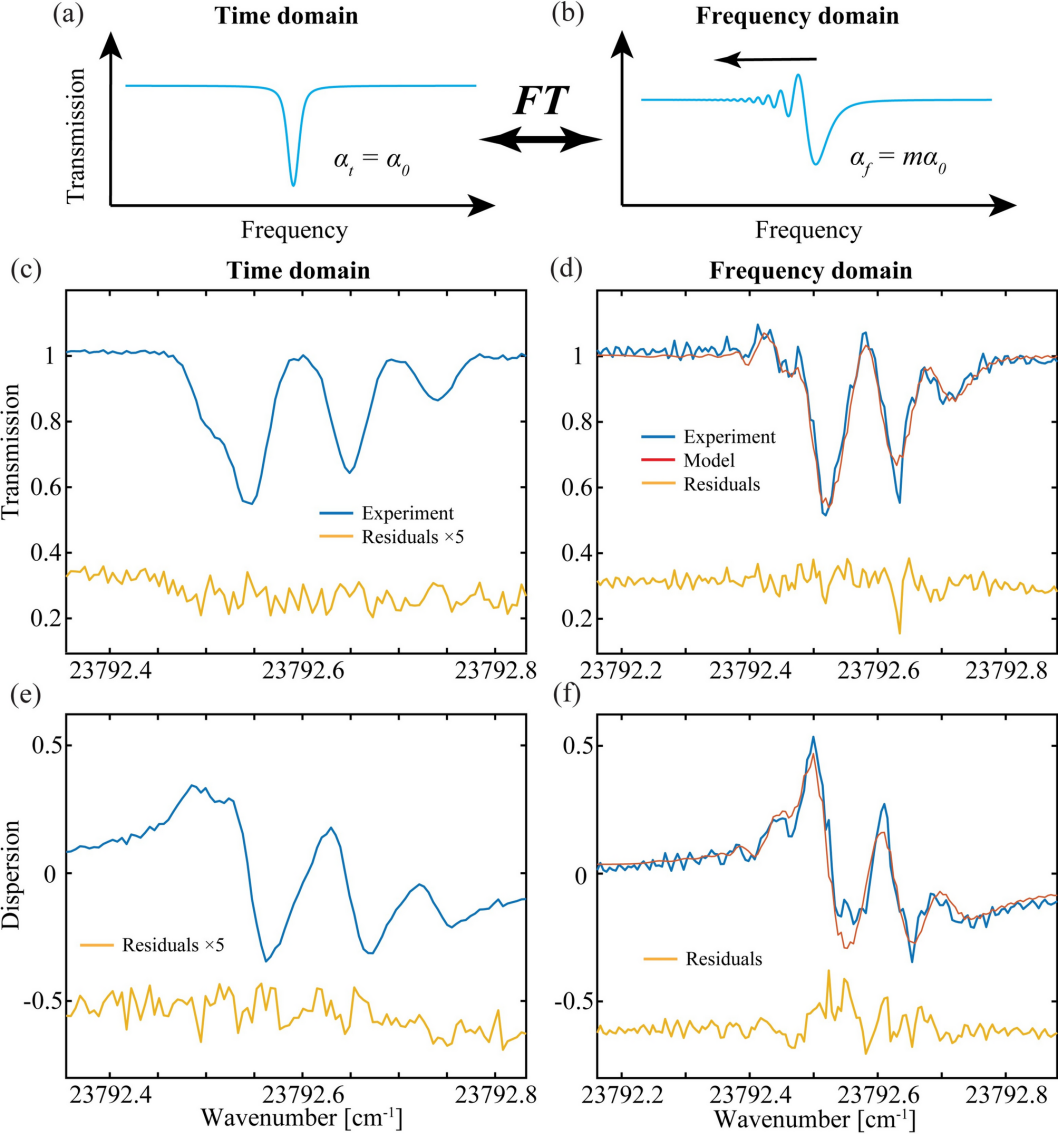
Rb SB results. (**a**) A model of time domain resonance that shows a symmetric line shape while in (**b**) the frequency domain model shows rapid passage in one direction. (**c**) The experimental time domain amplitude and (**e**) phase spectra of Rb atoms in blue, and the residuals (× 5) in yellow. The frequency domain (**d**) amplitude and (**f**) phase spectra in blue, the models in red and unscaled residuals in yellow.

**Fig. 4:**
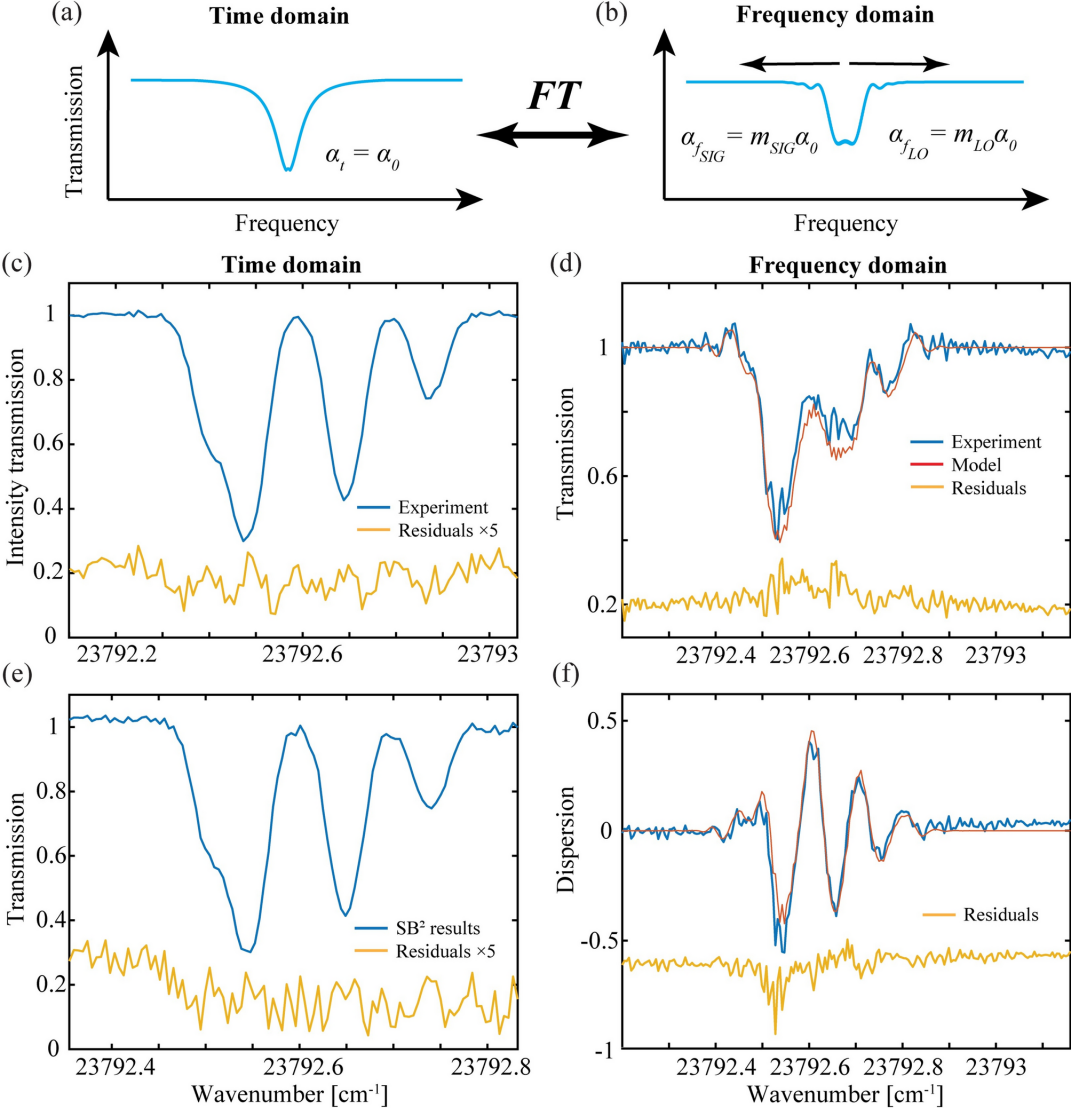
Rb CB results. Model predictions for the CB system that give (**a**) the intensity (squared magnitude) transmission spectrum in the time domain, and (**b**) the frequency domain spectrum where rapid passage effects are seen in both directions (see Methods for details). (**c**) The experimental intensity transmission spectrum of Rb is shown in the time domain (blue) with magnified residuals (× 5, yellow). (**d**) The observed (blue) and predicted (red) intensity transmission spectra and (**f**) the dispersion spectrum (red) are shown in the frequency domain with unscaled residuals (yellow). (**e**) For comparison, the squared magnitude of the SB data from Fig. 3(c) is shown with residuals against the squared magnitude of the model.

For both methods, the unmagnified time domain spectrum shown in Figs. 3a and 4a are recovered after inverse Fourier transformation of the frequency domain combs. The SB method samples the true magnitude and phase of the sample in both the time and frequency domains. The CB method samples the intensity (squared magnitude) spectrum, while in the frequency domain, magnification differences in the two directions creates a non-zero phase response that is the difference of the magnified LO and SIG phase spectra (current efforts are underway to recover phase).

## Results

### Separated beam (SB) results and analysis

We apply the SB method to measure the hyperfine absorption features of Rubidium (Rb) atoms near 420 nm in a 7.5 cm long cell that is insolated and heated to 113 C with heating elements on the cell and on each tilted window. The laser is locked at 11896.3523 cm^−1^ to center the near-UV comb near the Rb multiplet at 713.285 THz. The comb samples across the main four-line structure of Rb where each line consists of six hyperfine components^49^. The spectra span from 2 to 30 GHz on each sideband (where the time axis is converted to frequency using the SIG chirp rate). The SIG magnification factor for this data is *m*_*SIG*_ = 7499 from Eq. 11 for an optical LO bandwidth of 29.996 GHz and a RF bandwidth of 4 MHz. For the LO, *m*_*LO*_ = 7500 because the SIG optical bandwidth is 30 GHz.

With careful alignment of the two beams into the single mode fiber, we obtained about 15 million counts/s (CPS) at the PMT and nearly equal contributions from the SIG and LO legs. The second-order time and frequency domain amplitude spectra are shown in blue in Fig. 3c and d, respectively, and the corresponding phase spectra are shown in Fig. 3e and f. The fractional absorption of the amplitude transmission spectrum shown in blue is nearly 50%, and the rapid passage effects in the frequency domain are seen to extend only in one direction as predicted in Fig. 3b. The spectrum is fit by floating the magnitudes and positions of four lines (see Table I in Methods) using the measured temperature to fix the Gaussian line shape widths and vapor pressure of Rb atoms in the cell (the pressure broadening Lorentzian contribution is negligible). The scaled (× 5) residuals from the fits of the magnitude and phase spectra in the time domain show no clear structure above the noise and the magnitude spectrum has root-mean-square standard deviations (RMS SD) of 0.81% in Fig. 3c.

The best-fit time domain line shape in Fig. 3c is then used to predict the observed frequency domain profiles shown in Fig. 3d and f. The simulated sample spectra are mixed with the SIG chirped waveform in the frequency domain and are then down-converted by mixing with the LO in the time domain (see supplemental materials). The fits are in reasonable overall agreement with the observed spectra although the residuals are now shown unscaled.

### Combined beam (CB) results and analysis

With minimal change to the experimental alignment, we switched over to the CB method by changing a few fibers and by removing the final SIG/LO beam combiner in Fig. 1b. For these measurements, the laser frequency was locked to 11896.4835 cm^−1^. To make comparisons with the SB results, we attenuated the count rate on the PMT to about 11 million CPS. The second-order time and frequency domain spectra are shown in Fig. 4. The fractional absorption of the intensity (squared magnitude) transmission spectrum shown in blue in Fig. 4c now exceeds 70% in the time domain. The spectrum is fit by floating the intensities and positions of the four main features. The scaled (× 5) residuals indicate no apparent residual line shape errors that exceed the noise. For a SNR comparison, the square magnitudes of the SB data (blue) and residuals with the squared model (yellow) are shown in Fig. 4e. While the intensities are nearly identical to that of Fig. 4c, the residual’s RMS SD for the former is 1.52% (or 1.78% after correction for the different count rates) compared with 1.12% for the CB method. The improvement by ≈ 1.4 illustrates the sensitivity enhancement when acquiring the intensity spectrum using the CB method.

The best-fit time domain profile in Fig. 4c is then used to predict the observed frequency domain profiles shown in Fig. 4d and f. Unlike the SB method, this line shape is first mixed with both the SIG and LO chirped waveforms in the frequency domain and then mixed with the complimentary waveform in the time domain (see supplemental materials). This results in the predicted temporally magnified line shape functions shown in red in Fig. 4d and f with the residuals shown unscaled.

Interestingly, for both CB and SB data, the apparent SNR of the frequency domain is much worse than in the time domain. In contrast to other studies of ours where this same procedure was applied, the residuals in Figs. 3d and f and 4d and f show that the forward transformation of the best-fit time domain data does not fully model the observed responses in the frequency domain^42^. This suggests the higher frequency structure in the residuals is due to magnified spectral features. Additional spectroscopic information appears to be needed to properly account for this structure. Although not explored further here, the additional features may be associated with the unresolved hyperfine structure that makes up each of the four main features^49^.

### Measurements of the toxic pollutant, NO_2_

**Fig. 5:**
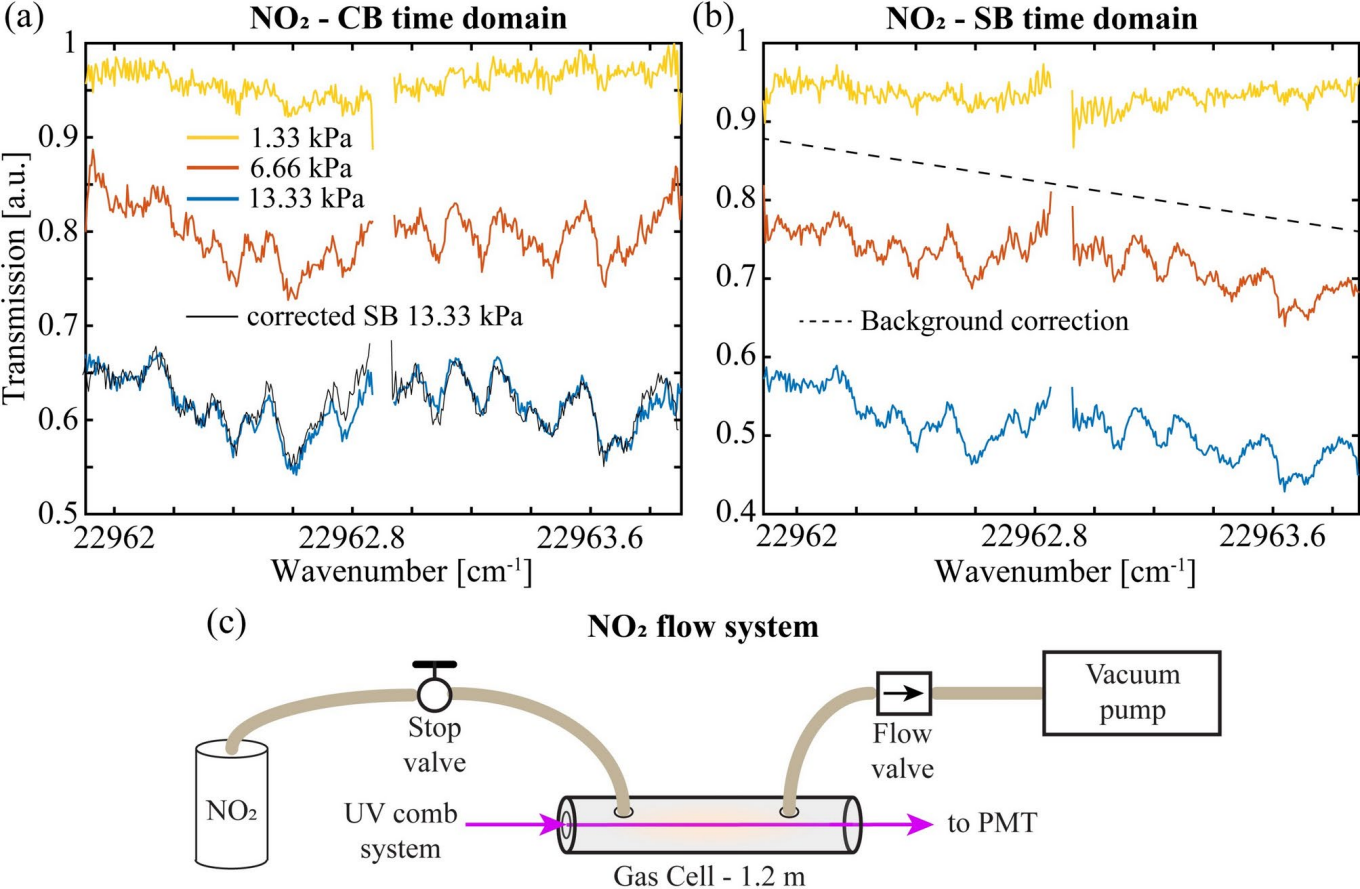
NO_2_ results. (**a**) The time domain CB and (**b**) SB squared magnitude spectra of a 2 cm^-1^ section of the broadband absorption band of NO_2_ near 435.5 nm obtained at three pressures of a 2000 ppm gas mixture. (**c**) The gas sample system for the absorption measurements consists of a 1.2 m long glass cell with wedged windows and flow regulators to maintain a fixed pressure of the 2000 ppm gas mixture.

The pollutant, nitrogen dioxide, is a common hazardous gas product of many combustion processes including gas stoves^50^ and is important in catalytic cycles that form and destroy ozone in the atmosphere^51,52^. Its room temperature spectrum in the near UV region spans from 415 to 525 nm and is highly congested and largely unresolved having feature widths near the Doppler limit of 1 GHz^53^. Both methods were used here to obtain a 2 cm^−1^ section of the spectrum near 435.5 nm in a 120 cm long cell with wedged windows in a flow configuration illustrated in Fig. 5c. The (time domain) results are shown in Fig. 5a and b at total pressures of 1.33 kPa (10 torr, yellow), 6.66 kPa (50 torr, red) and 13.3 kPa (100 torr, blue) of a 2000 ppm gas mixture. The spectra were ratioed against that of the evacuated cell.

The expected fractional absorptions at the above pressures are 0.95, 0.80 and 0.63, respectively^53^ and are in good agreement with the CB data shown in Fig. 5a. Alternatively, the squared magnitude of the SB spectra in Fig. 5b only compares well with the CB results following a background correction (black dashed line). In both cases, the high resolving power of the dual comb instrument reveals the partially resolved structure that provides a unique fingerprint of this pollutant devoid of interferents. Since our spectral resolution is much higher than that reported in the database^53^, the smoothed data at 13.3 kPa in Fig. 5a was used for model fits of the data at 6.66 kPa and 1.33 kPa. The uncertainties from the least squares fits are 133 Pa and 173 Pa, respectively, to give a modest detection sensitivity of ± 3 ppm m. For comparison, a non-dispersive infrared (NDIR) technique near 6.2 μm is reported in ref.^54^ with a sensitivity of 2.8 ppm in 1 min together with references to several other NDIR methods of comparable sensitivity. The most robust and sensitive methods are based on visible/UV absorption of lasers in enhancement cavities (± 40 ppt in 1 s in ref.^55^) or via laser induced fluorescence (LIF) detection (< ± 10 ppt in 1 min in ref.^51^). Further, unlike these current techniques, the CB method following an amplifier stage could be readily adapted as a remote sensing instrument for integrated path measurements of target gases^38^.

## Discussion

The advantages and disadvantages of the combined beam (CB) and separated beam (SB) methods demonstrated in this work are now enumerated to help guide which approach is best suited for a particular application. One area of particular interest is the ease of which beam overlap can be achieved to ensure the highest possible modulation depth (MD) at the detector. For a given count rate, the relative magnitude of the MD translates directly to the SNR of the comb lines since anything less than 100% just shifts the optical amplitude to the DC component of the Fourier transform (see Eq. 6). As we demonstrate here for the CB method, one way to achieve near 100% MD at the detector is to use the CB output from a PM fiber. Near perfect overlap at the detector was achieved even after frequency up-conversion in the crystal and the fiber coupling to the detector using a large 50 µm-core multi-mode (MM) fiber. The high efficiency of MM coupling led to PMT saturation with signal levels exceeding > 250 million CPS (i.e., the maximum count rate possible) and therefore, significant attenuation was required using neutral density filters to reduce rates for linear detection (10 million CPS to 50 million CPS). To achieve nearly 100% MD at the detector using the SB method required careful collimation of both beams into a 4 um-core SM fiber. Further, the efficiency of single mode (SM) fiber coupling of the two beams was strongly dependent on the matched wavefronts, focus, and the degree of spatial mode overlap of the coupled beam components. Since these conditions could only be partially achieved by compensation of the astigmatic UV beams using the cylindrical lens, the maximum count rate achieved for SM coupling of both beams was 20 million CPS. Initial attempts at SB coupling through 300 µm and 50 µm MM fibers also led to PMT saturation but at unacceptable low MDs of < 2% and < 25%, respectively.

A second important issue that differs for the two methods is the degree of phase instability since phase jitter at the detector over the averaging period results in a shift of optical amplitude to the DC component of the RF comb spectra. As discussed in Methods (Fig. 7), the phase instability of the CB method was shown to be about threefold smaller than the SB method. For the CB setup, instabilities primarily arise from the relative phase drift in the fibers between the SIG and LO legs of the interferometer (see Fig. 1a). The thermal and acoustic isolation of these fiber components led to a small and slow drift that was readily corrected for by the phase lock system^39^. The beat note signal for the lock is generated from the residual near-IR combined beam after up-conversion which, for ease of alignment, is coupled to the detector using a MM fiber with a 10 μm core. As in the UV, the MD in the near-IR was near 100%. We note that the faint UV signals from the PMT precluded their use in an active feedback loop for phase corrections. For the SB method, the interferometer optical path over which phase errors can occur is extended to include the free-space UV beam paths of the SIG and LO legs. Despite the stable single-crystal crossed-beam scheme used for UV generation, only after the UV beam path was fully covered were phase errors small enough not to wash out the spectrum (see supplemental materials section). Further, the sample itself can cause large phase fluctuations, where, for example, in an initial attempt, a temperature gradient between the windows and body of the Rb cell caused complete washout of the SB interferogram while the CB interferogram remained phase stable. Finally, the SB method required the careful collimation and overlap of the residual near-IR beams into the MM fiber, which further degraded the phase lock because of the reduced MD (< 50%) at the detector.

A third advantage of the CB system is in the simple implementation of a dual beam system where, a split off portion of the UV beam prior to the sample would probe a reference path for normalization. The dual beam method is advantageous where sample evacuation or detuning to an off-resonance region is not feasible. A corresponding dual beam approach for the SB method would require matching the path lengths for temporal chirped pulse overlap of the SIG and LO legs in both the sample and reference arms.

The SB method has a few distinct advantages over the CB method that may be of importance in certain specialized applications. Because the SIG and LO beams are combined after the sample, both the amplitude and phase spectra of a sample are recovered with similar SNR as shown in Fig. 3c and e. These independent measurements give insight into the complex behavior of light interactions in a sample and, because of the narrower line widths in the amplitude spectrum, enhance the resolving power. A second advantage is in the reduced number of mixing products in the up-conversion process. This process significantly increases the range of parameters available for different interleaving schemes that may be implemented to reach higher orders for increased optical bandwidth coverage and higher magnifications in the RF to optical conversion to reduce the detection bandwidth. In contrast, the RF comb spectrum of the CB method for a given down-conversion scheme has limited empty frequency space because of the presence of the interleaved degenerate combs. However, we found an insufficient increase in the SNR (counts per comb tooth) for the SB method relative to the CB method despite the former having far fewer comb teeth. This uniform SNR across different methods arises in part because of the undepleted pump from the low nonlinear mixing efficiency.

## Conclusions

In this paper, we have demonstrated two methods to generate near-UV combs that span up to 120 GHz (4 cm^−1^) by sum frequency generation in a single LBO crystal. Both methods rely on differential chirped pulse down conversion and interleaving of the EOM orders using a phase slip of the LO waveform to sample over a 4 kHz beat note in the near-IR.

In one method, the combined beam (CB) fiber-coupled output from the SIG and LO legs of the interferometer is focused inside the nonlinear crystal, generating an interferogram that contains a multitude of interleaved RF mixed combs, some of which retain a unique spectral map to the optical domain and some to degenerate combs that lack a unique one-to-one mapping. We compare the CB results to a more conventional separate beam (SB) method where each near-IR leg is first up-converted in crossed paths though a single crystal and then combined after sample interrogation. This crossed beam approach simplifies the phase matching conditions to a single phase-matching-angle adjustment. While the SB method always results in unique RF combs, both methods generate odd-order UV combs from mixing with the doubled carriers that pass through the EOMs. For both methods, high SNR comb spectra of the Rb isotopologues and unresolved hyperfine structure near 420 nm were obtained with RMS residual standard deviations near 1%. Further, because of the dual chirped-pulse down-conversion scheme, temporal magnification effects that are present only in the frequency domain are seen to distort the line shapes. However, the magnification has revealed additional high frequency structure that may be associated with the unresolved hyperfine components of the ^87^Rb and ^85^Rb isotopologues. The natural UV spectra devoid of these effects were easily recovered following the back-transformation to the time domain. From non-linear least squares fits of spectra obtained from both methods in the time domain, the best-fit frequencies and intensities of the four main-line features are in excellent agreement across four independent spectra obtained from the second- and third-order combs as evidenced by RMS residuals of ≈ 1%.

Finally, the advantages and disadvantages of the two methods are enumerated. The practical implementation of combining the near-UV beams in the SB method presents multiple challenges, making the CB method more favorable. Overall, the CB setup has advantages in (i) higher phase stability, (ii) higher throughput power to the detector, and (iii) the ease of alignment and use. On the other hand, the SB method (i) enables the acquisition of amplitude and phase spectra and (ii) has a far cleaner RF spectrum containing only uniquely mapped comb teeth.

## Methods

### Near-IR comb generation

The system shown in Fig. 1a is similar to that described to generate frequency combs in the THz region based on difference frequency generation^41^. As mentioned in this prior work, this same approach is applied here to instead generate UV combs via sum frequency generation. Briefly, a cavity stabilized Ti:Sapp ring laser (720 nm to 980 nm) pumped with 10 W of 532 nm light generates more than 250 mW of near-IR radiation. The laser is externally locked to a reference cavity to achieve a frequency stability of < 1 MHz in short term (< 10 s). A small portion is also split off by an optical wedge for wavemeter readout and for long-term drift control (< 2 MHz) using a HeNe stabilized reference cavity^56,57^.

The remaining output is free space coupled into a polarization-maintaining single mode fiber (PMSMF) with > 60% efficiency and split into the two legs of the interferometer that define the signal (SIG) and local oscillator (LO) combs. Each leg consists of an acousto-optic modulator (AOM, Brimrose, TEM-50-2-60-850-2FP, (5–6) dB insertion loss) followed by an electro-optic phase modulator (EOM, EO-space, PM-5SE-10-PFA-PFA-850-LV, 3 dB insertion loss). AOM1 and AOM2 are driven with function generators (FG, Keithley, 3390, amplified to 1 W) at 50.000 MHz and 49.996 MHz, respectively, to generate a 4 kHz beat note that separates the positive and negative EOM sidebands. The EOMs are driven by chirped pulses from an arbitrary waveform generator (AWG, Keysight M8195, 65 GS/s, 8-bit), each amplified by up to 1 W over the bandwidth from 1 to 15 GHz. The overall optical insertion loss for this system is ≈ 10 dB.

### UV generation

In the first arrangement shown in Fig. 1b referred as the combined beam (CB) method, the two near-IR legs are first combined in a 50/50 fiber coupler and one of output legs is collimated and focused inside the LBO crystal with a f = 5 cm achromatic lens. The sum frequency UV output having S-polarization is collimated with a second f = 5 cm lens, polarization rotated with a waveplate and spatially filtered from the residual fundamental following a Pellin Broca prism. The SIG + LO UV beams pass through a f = 30 cm cylindrical lens to correct astigmatism of the Brewster-cut crystal and then through a heated (113 °C) 7.5 cm long Rb reference cell equipped with tilted windows or a 1.2 m long glass cell with wedged windows for studies of NO_2_. The output beam is coupled into a multimode fiber (MMF, 50 μm core) for detection on a low-dark-count photomultiplier tube (PMT, EMI 9813QA, -1800 V, quantum efficiency of ≈ 25% from 190 to 450 nm). The typical near-IR power at the crystal is 5 mW to 10 mW and, while too low to be measured on a power meter, the conversion efficiency to the UV is expected to be near 0.01%^14,58^.

In the second arrangement, also shown in Fig. 1b and referred as the separated beam (SB) method, the SIG and LO UV beams are generated separately in a crossed path configuration in a single LBO crystal where the SIG beam travels in one direction through the crystal, while the LO beam travels in the opposite direction. A pair of dichroic mirrors (Thorlabs, DMLP505) on either side of the crystal are used to reflect the UV light (R > 99%) and pass the near-IR (T > 90%) to the crystal. Each leg is passed through a half waveplate and Pellin Broca prism to separate the residual near-IR. The SIG is sent through the Rb reference cell or NO_2_ cell before being overlapped on a 50/50 beam combiner with the LO. Only one of the outputs from the splitter is coupled into a SMF (Thorlabs, P5-305AR-2, 4 um core) and detected with the PMT system. The second output is currently discarded although a second PMT system could be used to enhance the signal-to-noise ratio (SNR) since the same information is contained in both halves except for a simple 180° phase shift. It is further noted that for the CB method, the discarded output from the final near-IR fiber combiner in Fig. 1b could also be frequency summed in the crossed beam configuration and simultaneously passed through the sample cell to improve the SNR again or to serve as the reference channel in a dual beam arrangement not implemented here.

For both the CB and SB methods, phase locking of the 4 kHz beat note in the near-IR was performed for coherent averaging over periods of up to 10 s using a scheme described elsewhere^39^. To achieve the best phase lock stability in the UV, the two residual near-IR beams reflected from the dichroic mirrors in Fig. 1b are combined on a polarization beam splitter, coupled into a 10 μm fiber and detected using a 200 MHz photodiode. The modulation depths (MDs) of the 4 kHz beat note typically ranged from 30 to 40%. For the CB method, the near perfect overlap in the PMSMF of the co-propagated SIG and LO beams resulted in a MD that was always near 100%.

The AWG and ADC systems are triggered at 800 Hz by a pulse delay generator (SRS, Model 535) where each interferogram consists of a 1 ms-long 1 megasample (MS) record for each of the counts and current channels. For both methods, the phase coherence is sufficiently long to coadd 4400 interferograms during 5.5 s in real time at 80% throughput, prior to storage on a hard disk. Typically, measurements were performed over a 15 min period where the final comb spectra were obtained from the average of the Fourier transforms (FTs) of each record (either counts or current). To remove standing wave effects arising from the Rb cell windows, background spectra are obtained by detuning the laser by (1–2) cm^−1^. The AWG, ADC and FGs are all disciplined using the same 10 MHz Rubidium clock reference.

For both methods, the PMT output(s) is first amplified by a high speed (τ ≈ 1 ns) low noise transimpedance amplifier (LNTA, Femto, HCA-400M-5K-C) and digitized at 1 gigasample/s (GS/s) in an analog-to-digital converter (ADC, Gage, CSE123G2, 12-bit) equipped with a field programmable gate array for data streaming to computer memory. For up to 2 channels, photon counting and photocurrent averaging are performed on the data streams, and the accumulated counts and average current records are stored to disk with a throughput of > 80% to enhance the dynamic range and to eliminate issues with pulse pile-up error. As we first described for a multi-heterodyne remote sensing system near 1.6 μm^38^, interferograms are formed by the accumulated counts as well as by the averaged photocurrent. Typical signal levels in this work range from 10 million counts/s (CPS) to 50 million CPS (dark count rates are < 50 CPS), the higher of which is the upper threshold where the pulse pile-up error begins to appear. Because of these high-count rates, the interferograms and comb spectra from the count and current records are essentially identical and therefore, used interchangeably in this work.

To significantly increase the UV output power for photodiode detection will require near-IR amplifiers prior to doubling and/or enhancement of the nonlinear conversion using resonant cavities^59^ or confinement in doped nonlinear optical fibers or waveguides^60,61^. The adaptation of these methods to integrated photonics will provide several advantages, i) portable devices should reduce system cost, ii) the confined area of the mode will increase the nonlinear conversion efficiency^62^, and iii), the short, integrated optical links will reduce phase instabilities^25^.

### Multi-wave nonlinear mixing

For the CB nonlinear mixing process, the first-order mixing products result from the six different waves that pass through the crystal. These input waves can be described at any given time by,

3 $${E}_{input}\left(t,\omega \right)={E}_{SIG}\left(\omega \pm {\omega }_{SIG}(t)\right)+{E}_{LO}\left(\omega \pm {\omega }_{LO}(t)+\Delta {\omega }_{AOM}\right)+{E}_{SI{G}_{0}}\left(\omega \right)+{E}_{L{O}_{0}}\left(\omega +\Delta {\omega }_{AOM}\right)$$

where ${E}_{SIG}\left(\omega \pm {\omega }_{SIG}(t)\right)$ are the two chirped sidebands generated by the SIG leg, ${E}_{LO}\left(\omega \pm {\omega }_{LO}(t)+\Delta {\omega }_{AOM}\right)$ are the corresponding LO chirped sidebands, and ${E}_{SI{G}_{0}}\left(\omega \right)$ and ${E}_{L{O}_{0}}\left(\omega +\Delta {\omega }_{AOM}\right)$ are the two residual carrier waves. The terms ${\omega }_{SIG}(t)$ and ${\omega }_{LO}(t)$ describe the chirps of the SIG and LO, respectively. The beat note from the difference in the frequency shifts of the two AOMs is added to the LO terms as $\Delta {\omega }_{AOM}$. When this near-IR light is incident on a detector, the mixing products consist of ($\ni$) RF combs of the positive and negative sidebands that are separated by the AOM beat note frequency,

4 $$LPF\left\{{E}_{input}{\left(t,\omega \right)}^{2}\right\}\ni {E}_{SIG}{E}_{LO}\left({\omega }_{RF}\left(t\right)-\Delta {\omega }_{AOM}\right)+{E}_{SIG}{E}_{LO}\left({-\omega }_{RF}\left(t\right)+\Delta {\omega }_{AOM}\right)$$

where $LPF$ is a low pass filter response, and ${\omega }_{RF}\left(t\right)={\omega }_{SIG}\left(t\right)-{\omega }_{LO}\left(t\right)$ is the down-converted difference frequency product sampled by the square law detector that maps the spectral information in the optical region to the RF region. The first and second terms in Eq. 4 are the positive and negative sideband combs, respectively.

In the LBO crystal, the sum frequency mixing process is modeled by a high pass filter of the squared input waves in Eq. 3 that produces many different combs corresponding to the different sum frequency wave interactions,

5 $${E}_{crystal}\left(t,2\omega \right)\ni HPF\{{E}_{input}^{2}\left(t,\omega \right)\}$$

The output of the nonlinear crystal will generate an array of combs that must map uniquely to reveal the underlying spectroscopy. There is a special class of interactions defined as second harmonic generation, where the two input waves in the nonlinear interaction are the same frequency. The few second harmonic generated terms are,

6 $${E}_{crystal}\left(t,2\omega \right)\ni {E}_{SIG}^{2}\left(2\omega \pm 2{\omega }_{SIG}(t)\right)+{E}_{LO}^{2}\left(2\omega \pm 2{\omega }_{LO}(t)+2\Delta {\omega }_{AOM}\right)+{E}_{SI{G}_{0} }^{2}\left(2\omega \right)+{E}_{L{O}_{0}}^{2}\left(2\omega \right)$$

where key to this unique mapping is the LO term that now has an AOM shift twice that of the fundamental comb described in Eq. 3. When these terms are incident on a square law detector, the RF output is,

7 $$LPF\left\{{E}_{crystal}^{2}\left(t,2\omega \right)\right\}\ni {E}_{SIG}^{2}{E}_{LO}^{2}\left(2{\omega }_{RF}\left(t\right)-2\Delta {\omega }_{AOM}\right)+{E}_{SIG}^{2}{E}_{LO}^{2}\left(-2{\omega }_{RF}\left(t\right)+2\Delta {\omega }_{AOM}\right)$$

Equation 7 is nearly identical to Eq. 4, except the comb has twice the optical and RF bandwidth (and twice the number of comb lines). Further, these combs have twice the AOM shift so they are spectrally separated from the fundamental comb.

For both methods, there are two other combs generated by the sum frequency generation process that form a comb at twice the AOM beat note separation. The products between the chirp and its corresponding carrier are,

8 $${E}_{crystal}\left(t,2\omega \right)\ni {E}_{SIG}{E}_{SI{G}_{0}}\left(2\omega \pm {\omega }_{SIG}(t)\right)+{E}_{LO}{E}_{L{O}_{0}}\left(2\omega \pm {\omega }_{LO}(t)+2\Delta {\omega }_{AOM}\right)$$

As before, these terms mix on the detector to produce a comb that spans the same amount of bandwidth as the fundamental comb but have a unique mapping in the near-UV spectrum due to the AOM beat note doubling of the carrier.

9 $$LPF\left\{{E}_{crystal}^{2}\left(t,2\omega \right)\right\}\ni {E}_{SIG}{E}_{SI{G}_{0}}{E}_{LO}{E}_{{LO}_{0}}\left(\pm {\omega }_{RF}(t)\mp 2\Delta {\omega }_{AOM}\right)$$

This represents a mixed order comb, generated from the product of the first-order chirp and carrier wave.

We also find that at comparable count rates, the SNR of the SB comb with far fewer comb lines shows a lower-than-expected improvement relative to the CB comb. While the SB interferogram in Fig. 2c was acquired at a 35% higher count rate (15 MCPS vs 11 MCPS for the CB method), the SB comb spectra in Fig. 2d and e show only between 3 to 5 dB higher SNRs, respectively, which are not enough to account for the very strong degenerate combs shown in Fig. 2c. Part of this is rationalized because of the low crystal mixing efficiency in single pass (≈ 0.01%) where the absence of pump wave depletion leads to fixed powers in each unique comb. Further, the integrated power in the DC component of the interferograms from Eq. 6 is found to be reduced by more than twofold for the CB method where the degenerate combs must receive some power to account for the similar count rates observed for the two methods. Figure 2d shows the first-order comb, where the comb teeth are at twice the AOM frequency difference for both system designs. It follows that any combination of higher EOM orders will mix to generate UV combs. The drive voltage can be easily adjusted in the AWG to optimize for higher orders at drive voltages above *V*_*π*_ (≈ 3 V) of the modulators. The distribution of near-UV comb amplitudes is proportional to the square of the product of the Bessel function amplitudes. The third-order comb shown in Fig. 2c spans from 9 to 15 MHz and is produced from the product between the first- and second-order fundamental combs.

### Data processing

The down-converted optical frequency comb spectra shown in Fig. 2c, d and e are obtained from the 15 min average of the Fourier transforms of multiple records like that shown in Fig. 2a. The comb teeth obtained for the Rb spectrum near 420 nm are normalized against the background comb to produce the frequency-domain spectrum. Prior to normalization, the selected comb teeth of a given order are inverse Fourier transformed then normalized to generate the corresponding time domain spectrum. Due to the quadratic phase imparted onto the system response by the chirped LO pulse, the transient response is magnified in the frequency domain^39^. A further complication occurs for the CB method where both the SIG and LO sample the Rb spectrum and then act to magnify each other, resulting in two different magnification factors of opposite sign (i.e., direction),

10 $${m}_{SIG/LO}=\frac{\Delta {f}_{LO/SIG}}{\Delta {f}_{SIG/LO}-\Delta {f}_{LO/SIG}}=\pm \frac{\Delta {f}_{LO/SIG}}{\Delta {f}_{RF}}$$

where *m*_*SIG*_ and *m*_*LO*_ are the magnification factors of the SIG and LO legs, respectively. The frequency domain spectra experience a magnified chirp rate, ${{\alpha }_{f}}_{SIG/LO}$, where the temporal response is ${m}_{SIG/LO}$ times faster than the natural chirp rate, ${\alpha }_{SIG/LO}$,

11 $${{\alpha }_{f}}_{SIG/LO}={m}_{SIG/LO}{\alpha }_{SIG/LO}$$

The inverse Fourier transform removes the quadratic phase response imparted on the frequency domain line shape to give the unmagnified (natural) spectrum. When the chirp rate in the frequency domains exceeds the response time of the system (which is proportional to the spectral width), the rapid passage effects will cause oscillations (ripples) that distort the symmetric line shape (oscillations may even be observed in the time domain for even faster chirps that were not performed here)^40^. Figures 3b and 4b show the SB and CB model predictions of a single resonance in the frequency domain where these ripples are observed in one direction only or in both directions, respectively. For the SB method, the single-ended magnification arises from the LO quadratic phase alone in contrast to the CB method where complimentary magnifications from Eq. 11 occur in both directions by the simultaneous passage of the SIG and LO through the sample^25^.

### Bandwidth expansion

**Fig. 6:**
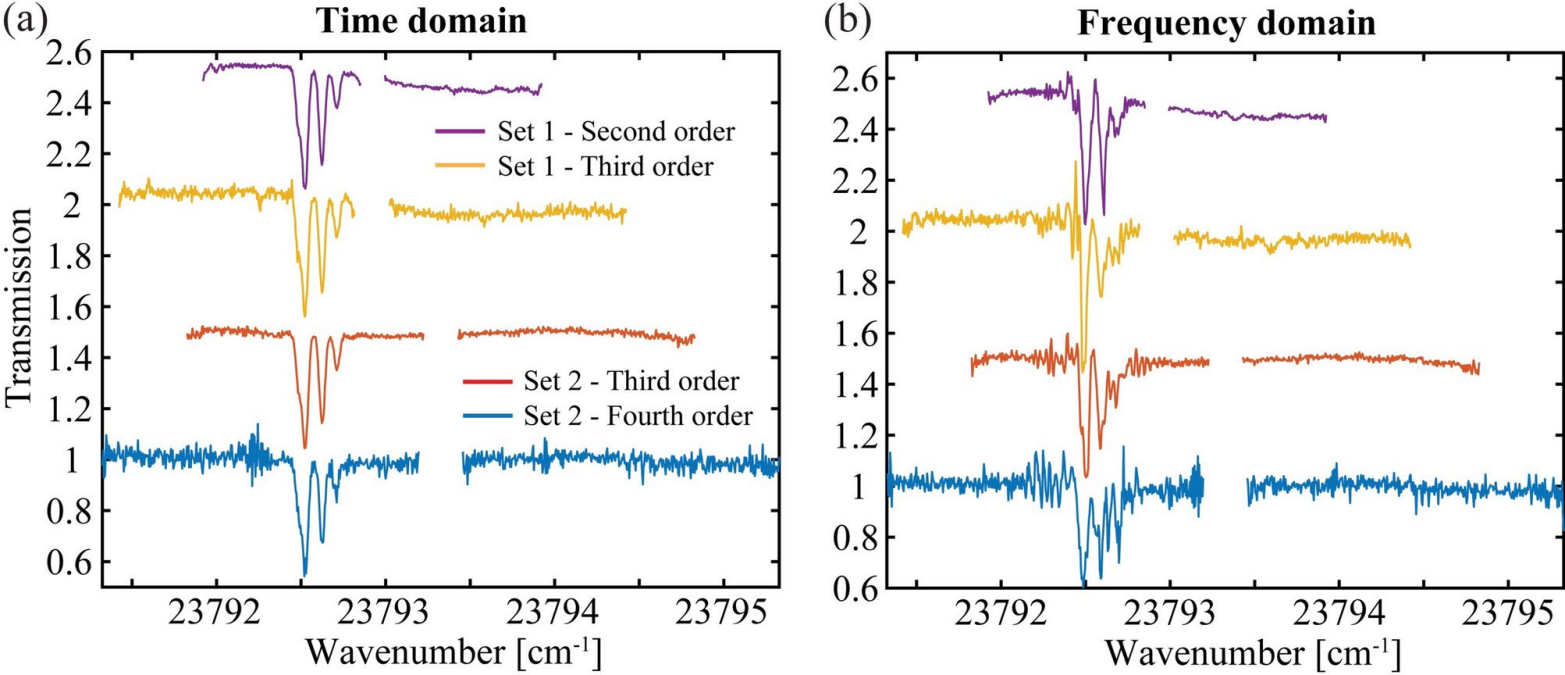
Bandwidth expansion. (**a**) The time domain amplitude spectra and (**b**) frequency domain spectra of two data sets showing the bandwidth expansion form second order to the fourth order.

The drive voltage on the AWG is increased to generate higher-order outputs from the EOMs in the near-IR and as discussed earlier, for the higher-order mixed combs in the UV. This control allows for a trivial way to expand the bandwidth of the system. Figure 6 shows the time and frequency domain amplitude transmission spectra of two sets of data acquired with the SB method. For the first data set, the second- and third-order amplitude transmission spectra are shown as the purple and orange traces in Fig. 6 for the span 2 cm^−1^ and 3 cm^−1^, respectively (the second-order spectrum is the same as in Fig. 4). For the second data set, the drive voltage is increased by ≈ 30%, and the laser frequency is adjusted to 11896.5548 cm^−1^ to better center the Rb profile in the negative sideband region. The third-order and fourth-order spectra are shown as the red and blue traces in the lower sections of Fig. 6, respectively. First notice that the width of the center gap region increases with the bandwidth of the order. Further, as the chirp rate is varied and since the magnifications in the frequency domain depend on order, the magnitudes of the ripples are seen to increase with order in Fig. 6b. However, these effects are completely absent in the back transformed spectra shown in Fig. 6a. While the bandwidth coverage of the second-order comb is more than sufficient to measure the Rb absorption feature at 420 nm, the fourth-order comb gives an increased spectral coverage of nearly 4 cm^−1^ (in blue) We have also previously demonstrated fourth-order comb generation in the near-IR, which, for this method, would translate to an eighth-order UV comb^39^. While this work is mainly centered in the near-UV region, we expect a similar performance of the system components down to 360 nm with a simple change of the optics set in the Ti:Sapp laser and with the substitution of a second LBO crystal that is center-cut for 770 nm. With power amplification of the current system, non-linear mixing into deeper UV regions near 250 nm will be possible with the aid of enhancement cavities. This expanded UV region will enable the sensing of other toxic gases such as formaldehyde^63^, sulfur dioxide, and ozone.

### Fit results of the Rb isotopologues

The uncertainties of the fit parameters were estimated from the scatter in the repeated measurements of the fitted parameters across several data sets. The data sets included the two second-order spectra discussed above for the SB and CB methods and two additional fits of the third-order spectra obtained at the higher drive voltage in Fig. 6a. The average parameters and uncertainties (Type B, k = 1 or 1σ) of the line centers and intensities of each of the four features in the Rb spectrum are given in Table 1. The precision of the line centers is <± 20 MHz for the ^87^Rb transitions and <± l0 MHz for the ^85^Rb transitions. The absolute frequencies are estimated to be within ± 100 MHz based on the wavemeter reading following calibration with two polarization stabilized HeNe lasers (± 50 MHz). The precisions of the line intensities are < 4%. The uncertainties do not include the propagated errors from uncertainties in the cell temperature and cell path length although we estimate changes to be <± 1% over the period of these measurements.

**Table 1:** Best-fit line frequencies and intensities of the 6P_3/2_ ← 5S_1/2_ transitions of the ^87^Rb and ^85^Rb isotopologues.

Ground state	Frequency (THz)	Intensity (cm^−1^/mol cm^−2^ × 10^–16^)
^87^Rb F′′ = 2	713.281618 (15)	3.07 (3)
^85^Rb F′′ = 3	713.282791 (7)	7.18 (28)
^85^Rb F′′ = 2	713.285883 (7)	5.25 (12)
^87^Rb F′′ = 1	713.288586 (19)	1.80 (7)

Uncertainties shown in the least significant digits are determined from the RMS differences (Type B, k = 1 or 1σ) in four independent spectral fits of the four main-line features (see text for details).

### Comparison of the phase instabilities of the SB and CB methods

**Fig. 7:**
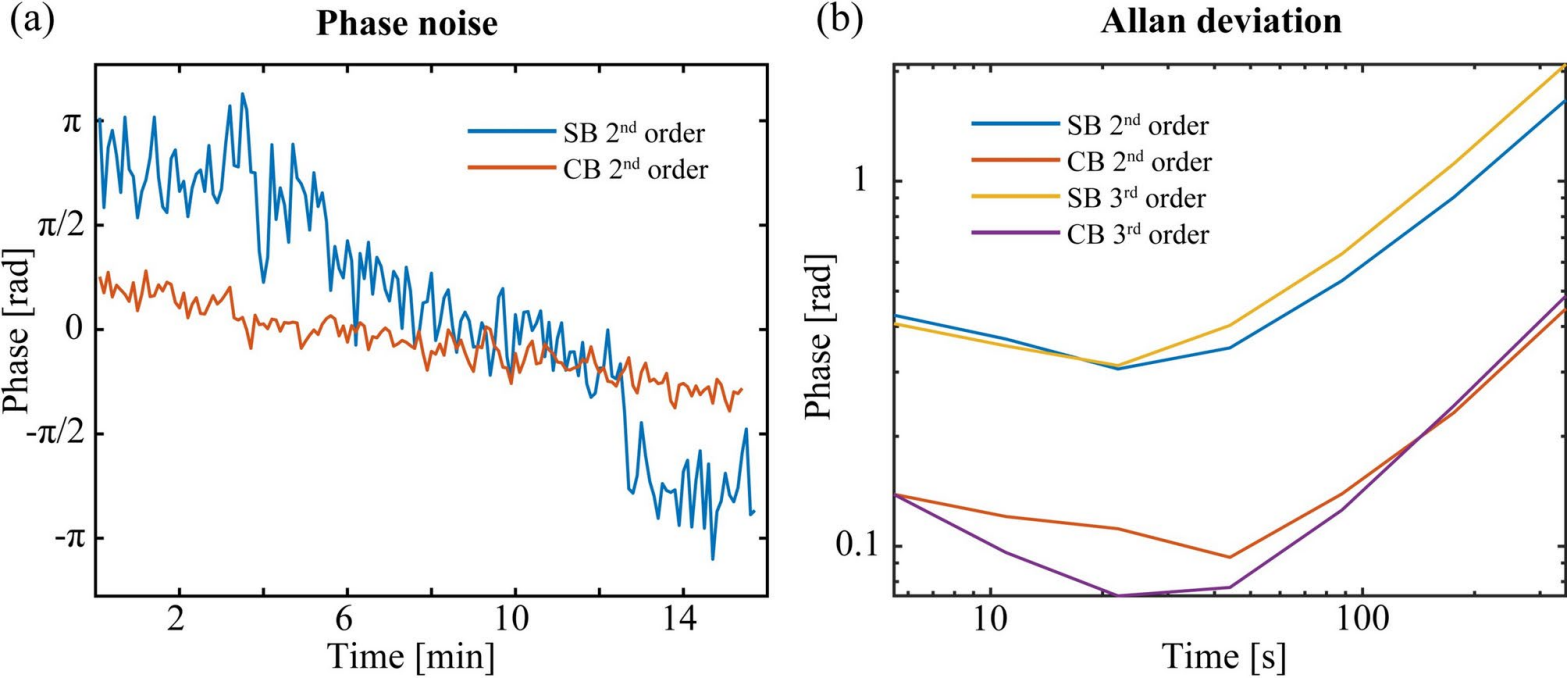
Phase noise. The average phase angle of the second order comb teeth as a function of time for the SB method, in blue and CB case, in red. (**b**) The Allan deviation plots of the four data sets, showing the significant short- and long-term improvement in the phase stability using the CB system.

The phase instability of the two methods was evaluated using the raw data records acquired on 5.5 s intervals which corresponds to 4.4 s records at 80% throughput. To illustrate the actual phase drift in the UV interferogram, the average phase angle of the comb teeth (8 kHz beat note) in the second and third orders are compared. The results are shown as a linear function of time for both methods in Fig. 7a. It is clear from the SB results shown in blue that this configuration suffers from long-term phase drift approaching 2π rad over the 15 min interval relative to a drift near 2 rad for the CB method, a nearly threefold improvement. The Allan deviation analysis shown in Fig. 7b substantiates this conclusion and additionally shows that the phase instability of the CB method is also ≈ threefold lower than that of the SB method. The upward turn in the phase deviation of the CB method suggests that signal averaging would be possible over 60 s intervals without any significant degradation of the MD. The longer path length of the NO_2_ gas sensing system shown in Fig. 5c increased the phase instability of the SB system, resulting in larger background fluctuations that decreased the sensitivity of the system. To partly alleviate these effects, the gas cell and free space beam paths were isolated from the room air fluctuations to minimize index variations and the near-IR connecting fibers to the doubling crystal were insulated with a foam wrapping. The added phase noise from these effects will need to be minimized for this method to be viable as a gas sensing system.

## Supplementary Information

Supplementary Information 1.

Supplementary Information 2.

Supplementary Information 3.

## Data Availability

Data is available at the following public repository: 10.18434/mds2-3832. The datasets generated during and/or analysed during the current study are available from the corresponding author on reasonable request. Certain commercial equipment, instruments, or materials are identified in this paper in order to specify the experimental procedure adequately. Such identification is not intended to imply recommendation or endorsement by NIST, nor is it intended to imply that the materials or equipment identified are necessarily the best available for the purpose.
