# Supplementary figures and images for: Frequency-doubled chirped-pulse dual-comb generation in the near-UV: combined vs separated beam investigations of Rb atoms and NO2 near 420 nm
Source: Sci Rep. 2025 May 25;15:18154. doi: 10.1038/s41598-025-00684-1 (PMC12104465; doi:10.1038/s41598-025-00684-1)

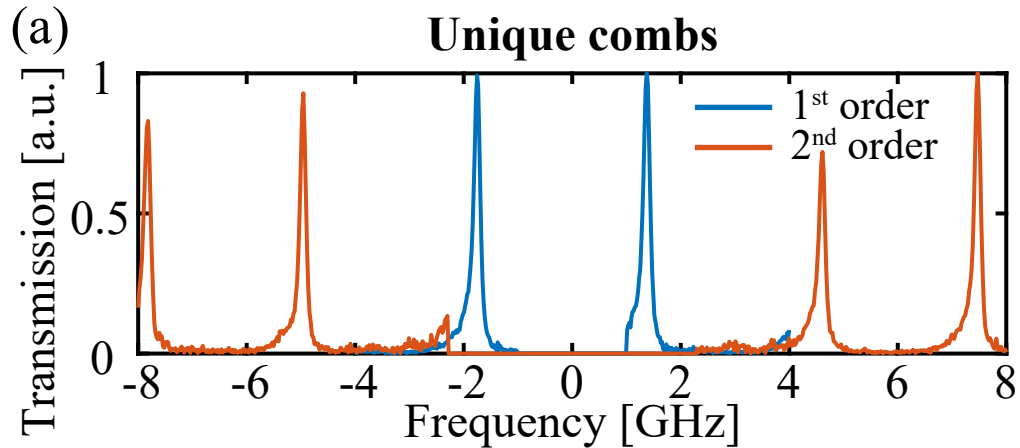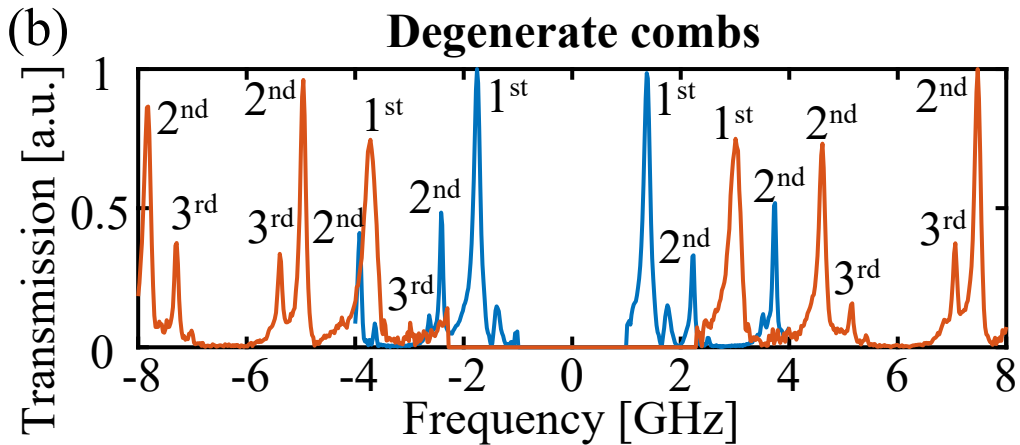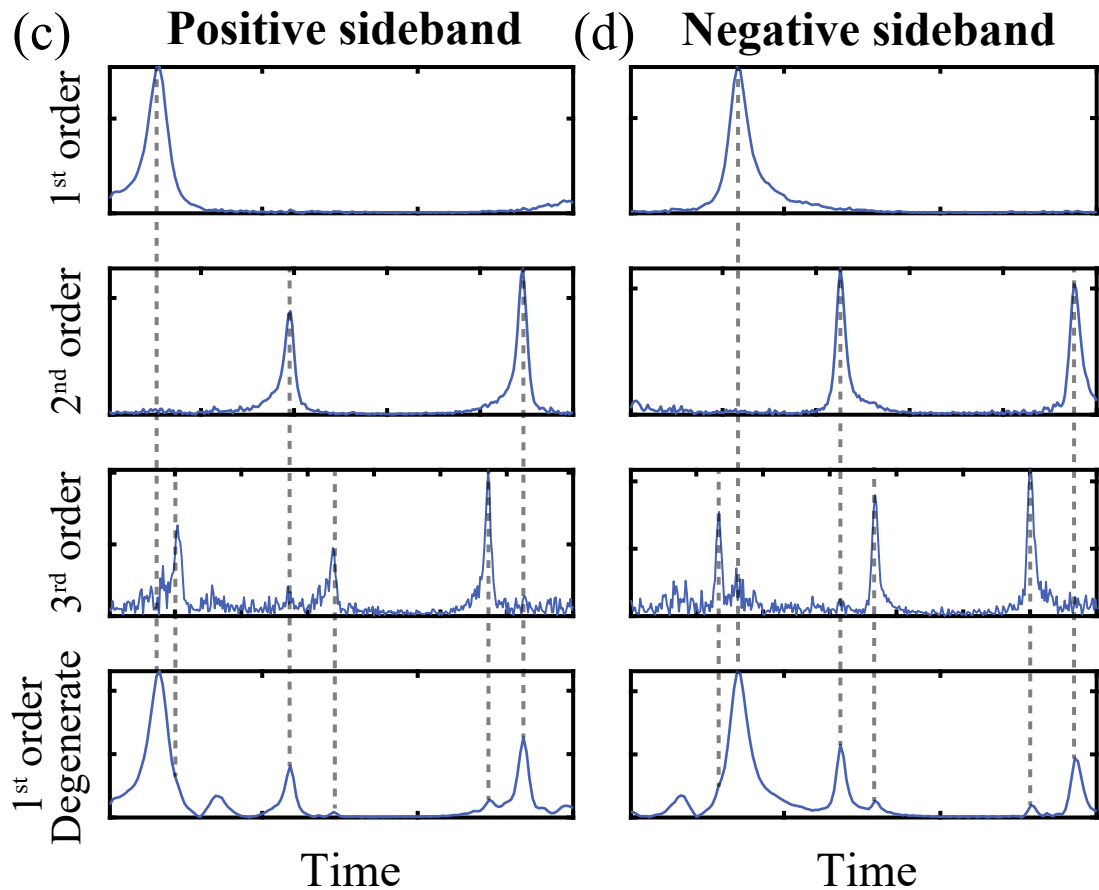

Supplement: Supplementary file 1 — Supplementary Information 1. [file 41598_2025_684_MOESM1_ESM.pdf]

# Unlocked phase system

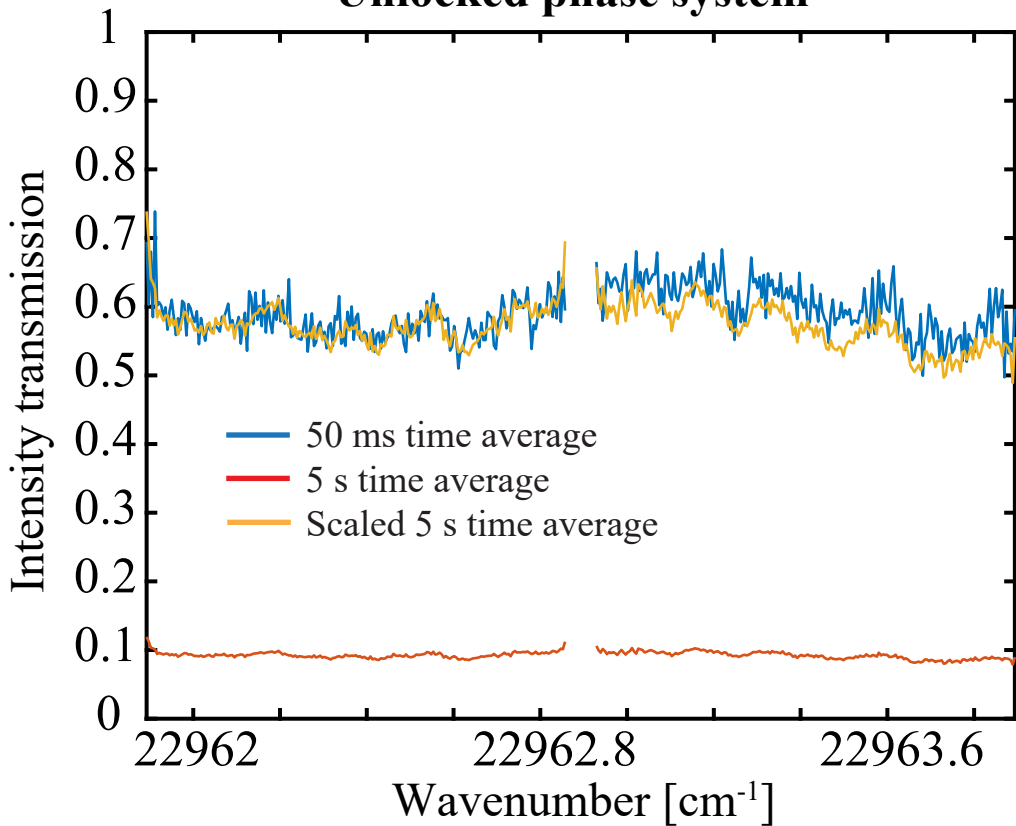

Supplement: Supplementary file 2 — Supplementary Information 2. [file 41598_2025_684_MOESM2_ESM.pdf]
