# Supplementary material for: Frequency-doubled chirped-pulse dual-comb generation in the near-UV: combined vs separated beam investigations of Rb atoms and NO2 near 420 nm
Source: Sci Rep. 2025 May 25;15:18154. doi: 10.1038/s41598-025-00684-1 (PMC12104465; doi:10.1038/s41598-025-00684-1)
Supplement: Supplementary file 3 — Supplementary Information 3. [file 41598_2025_684_MOESM3_ESM.docx]

Frequency-doubled chirped-pulse dual-comb generation in the near-UV: Combined vs separated beam investigations of Rb atoms and NO_2_ near 420 nm: Supplemental document

Jasper R. Stroud,^1,*^ and David F. Plusquellic^1^

^1^National Institute of Standards and Technology, Boulder, CO 80305

*jasper.stroud@nist.gov

1. Degenerate comb spectra

The terms in the combined beam (CB) architecture that form the degenerate combs are the mixing products between the SIG and LO chirps. Unlike the unique combs that are separated by twice the AOM beat note, degenerate combs form at both a single AOM beat note shift and no beat note shift. The sample that best illustrates this effect is an etalon in transmission mode that produces a sparse and repetitive spectrum. The etalon under test has a 3 GHz free spectral range (FSR) and a finesse near 30 resulting in about 100 MHz full width half max (FWHM) spectral features. The comb used to probe the etalon has a SIG chirp that spans from *f_start_* = 1 GHz to *f_stop_* = 4 GHz, with the other comb parameters identical to those described in the main text. The unique comb spectra for the first and second order combs are shown in Fig. S1a, in blue and red, respectively. With a 3 GHz FSR, we can see two resonance peaks in the first order, and four more in the second order, that spans 16 GHz with a 4 GHz gap. However, the degenerate combs illustrated in Fig. 2d in the main text and shown in Fig. S1b are a combination of the many different chirped comb spectra. The relationship is clear from the plots in Figs. S1c and S1d where the first, second, and third order comb spectra can be combined to form the degenerate combs seen in in the bottom panel in Figs. S1c and S1d. The labels in Fig. S1b show what order the spectral feature originated. We are currently working on techniques to deconvolve this spectrum to take advantage of the extra power put into these comb teeth compared to the separated beam (SB) system.


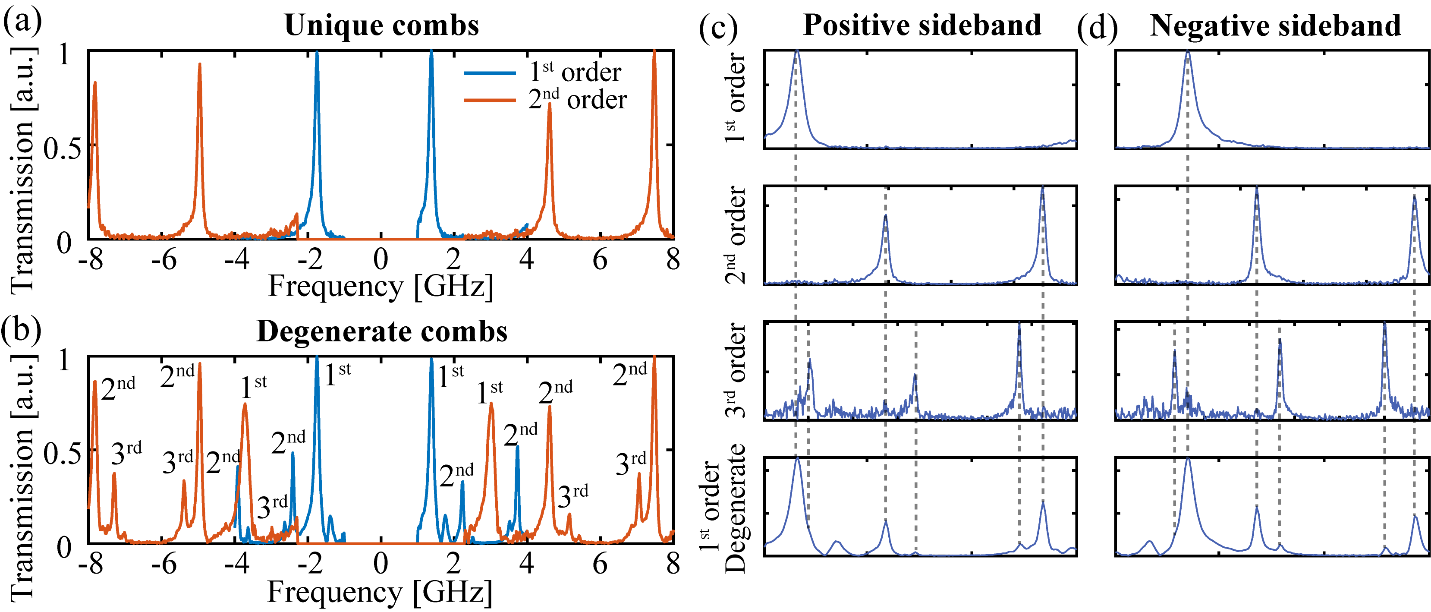


Fig. S1. UV spectra of the (a) 1^st^- and 2^nd^-order unique combs, (b) higher order degenerate combs and (c) the unique positive and negative sidebands that make up the degenerate combs (bottom). All spectra were obtained from the transmission spectrum of an etalon with a FSR=3 GHz and finesse of 30 (100 MHz FWHM).

1. Differential chirp down conversion

The model for the magnified frequency domain spectra is based on the fit parameters from the time domain data. The line shape model is used to translate the time domain parameters into the frequency domain line shape using the optical chirps that simulate the down conversion process into the radio frequency (RF) domain. The SIG and LO chirp pulses can be described by their quadratic phase terms that define the chirps,

$C_{SIG}\left( t \right)=\exp\left( i\pi\frac{\Delta f_{SIG}}{\tau_{CP}}t^{2} \right)$ (S1)

$C_{LO}\left( t \right)=\exp\left( -i\pi\frac{\Delta f_{LO}}{\tau_{CP}}t^{2} \right)$ (S2)

where $\tau_{CP}$ is the chirp duration and $\Delta f_{SIG}$ and $\Delta f_{LO}$ are the chirp ranges of the SIG and LO, respectively. The transfer function of the sample is the unconverted line shape model from the time domain fits that mixes with the SIG chirp in the frequency domain,

$E\left( \omega\right)=S(\omega)C_{SIG}\left( \omega\right)$ (S3)

where *S(ɷ)* is the spectral response of the sample. To down convert the SIG chirp into the radiofrequency domain, the sample output mixes with the chirped LO, which is the convolution between the output, Eq. S3, and the Fourier transform of the LO chirped pulse, Eq. S2.

$I\left( \omega\right)=C_{LO}\left( \omega\right)*E\left( \omega\right)=F\left\{ C_{LO}\left( t \right)F^{-1}\left\{ E\left( \omega\right) \right\} \right\}$ (S4)

where *F*{} is the Fourier transform operator. The frequency domain spectrum represented in Eq S4 shows how the LO chirp magnifies the sample spectrum in the frequency domain,

$I\left( \omega\right)=F\left\{ \exp\left( -i\pi\frac{\Delta f_{LO}}{\tau_{CP}}t^{2} \right)F^{-1}\left\{ E\left( \omega\right) \right\} \right\}$ (S5)

while in the time domain the quadratic phase shift from the LO is just a phase term and does not magnify the features in the sample spectrum.

$I\left( t \right)=\exp\left( -i\pi\frac{\Delta f_{LO}}{\tau_{CP}}t^{2} \right)E\left( t \right)$ (S6)

giving the natural time domain response of the sample under test.

In the CB system, the output is the product of the output described in Eq S5 and S6 with the corresponding mixing of the LO chirp and the sample, then down conversion with the SIG chirp.

$E\left( \omega\right)=S\left( \omega\right)C_{SIG}\left( \omega\right)+S(\omega)C_{LO}\left( \omega\right)$ (S7)

Then the frequency domain put is,

$I\left( \omega\right)=F\left\{ \exp\left( -i\pi\frac{\Delta f_{LO}}{\tau_{CP}}t^{2} \right)F^{-1}\left\{ S\left( \omega\right)C_{SIG}\left( \omega\right) \right\}+\exp\left( -i\pi\frac{\Delta f_{SIG}}{\tau_{CP}}t^{2} \right)F^{-1}\left\{ S\left( \omega\right)C_{LO}\left( \omega\right) \right\} \right\}$ (S8)

or in the time domain,

$I\left( t \right)=\exp\left( -i\pi\frac{\Delta f_{LO}}{\tau_{CP}}t^{2} \right)\left( S\left( t \right)*C_{SIG}\left( t \right) \right)+\exp\left( -i\pi\frac{\Delta f_{SIG}}{\tau_{CP}}t^{2} \right)\left( S\left( t \right)*C_{LO}\left( t \right) \right)$ (S9)

The detected signal is the product of these two terms in Eq S8 and S9, where the SIG and LO chirps combine to give the intensity spectrum in the time domain. In the frequency domain, the sign difference between the SIG and LO magnification results in a frequency domain phase spectrum that is the difference between the phase spectrum of the SIG and LO magnified phase data.

1. Unlocked phase data processing

The phase instabilities that plague the SB system result in the beat note deconstructivly interfering as the two optical paths of the SIG and LO leg vary with temperature and pressure. The small and slow fluctuations can be fixed by locking schemes, but the UV portion of the system is not compensated for resulting in some washout over the 5 sec averaging time. When the AOM beat note is unlocked, the phase noise nearly washes out the temporal interferogram completely over the 5 s average. Figure S2 shows a 10.6 kPa NO_2_ spectra obtained when the AOM beat note lock was turned off, resulting in uncorrected phase fluctuations that washed out the averaged interferogram. The data was acquired in two ways, first, as in the main manuscript, the data was averaged over 4400 records and saved in time every 5 seconds. Second, the same data was averaged over 44 records every 50 ms, then the Fourier transformed to be incoherently averaged over the magnitude and phase of 100 comb spectra to match the same total 5 second resolution. The 50 ms spectra shown in blue have increased noise content compared to the 5 s spectra in red due to the benefit of beating down of the noise in the time domain. However, the washout of the interferogram over the 5 seconds results in a large background offset, making it difficult to determine the actual absorption strength of the sample. The spectrum in blue shows the correct absorption strength because the Fourier transform was done in times shorter than the coherence time of the unlocked AOM beat note, minimizing washout. The trace in yellow shows the 5 s average scaled by 6.2 to approximately match the blue spectra, revealing the NO_2_ spectral features.


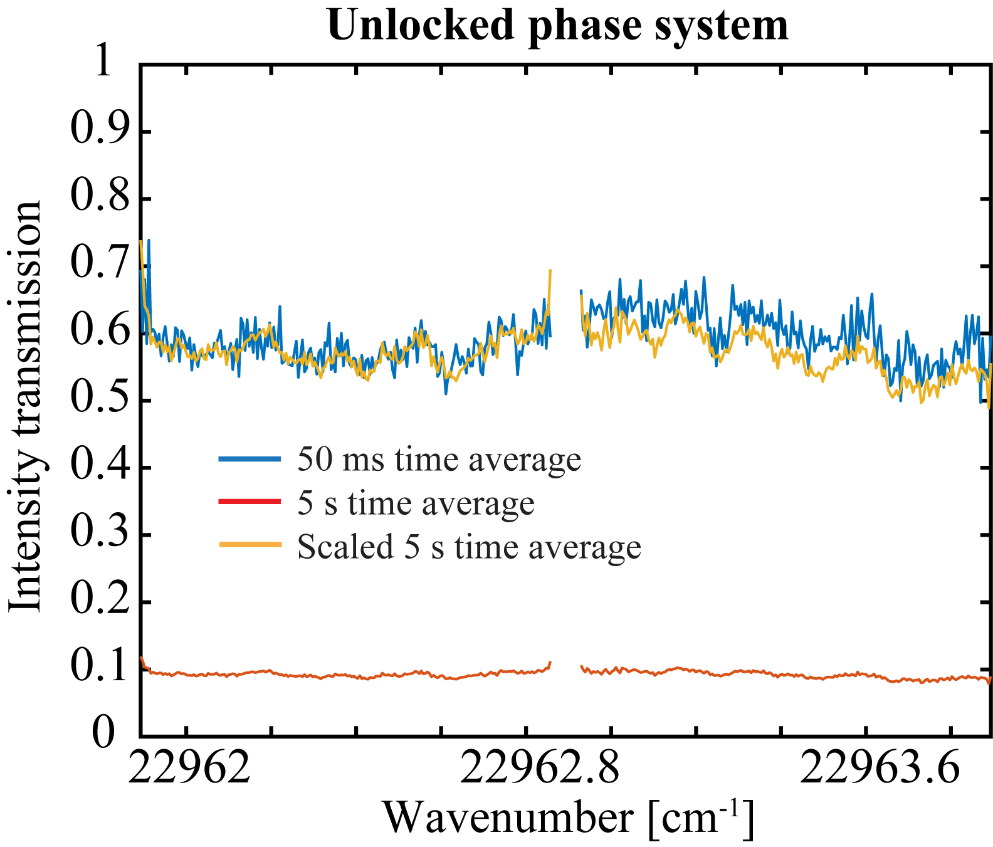


Fig. S2. The results when the system was not phase locked for Fourier transform done at a 50 ms time average in blue, and at a 5 sec time average shown in red and scaled by 6.2 in yellow (see text for details).
